# A scheme for a flexible classification of dietary and health biomarkers

**DOI:** 10.1186/s12263-017-0587-x

**Published:** 2017-12-12

**Authors:** Qian Gao, Giulia Praticò, Augustin Scalbert, Guy Vergères, Marjukka Kolehmainen, Claudine Manach, Lorraine Brennan, Lydia A. Afman, David S. Wishart, Cristina Andres-Lacueva, Mar Garcia-Aloy, Hans Verhagen, Edith J. M. Feskens, Lars O. Dragsted

**Affiliations:** 1 Department of Nutrition, Exercise and Sports, University of Copenhagen, Copenhagen, Denmark; 2 Department of Food Science, University of Copenhagen, Copenhagen, Denmark; 3 Biomarkers Group, Nutrition and Metabolism Section, International Agency for Research on Cancer (IARC), Lyon, France; 4 Agroscope, Federal Office of Agriculture, Berne, Switzerland; 5 University of Eastern Finland, Kuopio, Finland; 6 INRA, Human Nutrition Unit, Université Clermont Auvergne, INRA, F63000 Clermont-Ferrand, France; 7 UCD Institute of Food & Health, UCD School of Agriculture and Food Science, University College Dublin, Dublin, Ireland; 8 Division of Human Nutrition, Wageningen University & Research, Wageningen, The Netherlands; 9 Department of Biological Sciences, University of Alberta, Edmonton, Canada; 10 Biomarkers and Nutrimetabolomic Laboratory, Department of Nutrition, Food Sciences and Gastronomy, University of Barcelona, Barcelona, Spain; 11 CIBER de Fragilidad y Envejecimiento Saludable (CIBERFES), Instituto de Salud Carlos III, Barcelona, Spain; 12 European Food Safety Authority (EFSA), Parma, Italy; 13 University of Ulster, Coleraine, Northern Ireland UK

**Keywords:** Biomarker, Classification, Nutrition, Ontology, Exposure, Effect, Susceptibility, Metabolomics, Review

## Abstract

Biomarkers are an efficient means to examine intakes or exposures and their biological effects and to assess system susceptibility. Aided by novel profiling technologies, the biomarker research field is undergoing rapid development and new putative biomarkers are continuously emerging in the scientific literature. However, the existing concepts for classification of biomarkers in the dietary and health area may be ambiguous, leading to uncertainty about their application. In order to better understand the potential of biomarkers and to communicate their use and application, it is imperative to have a solid scheme for biomarker classification that will provide a well-defined ontology for the field. In this manuscript, we provide an improved scheme for biomarker classification based on their intended use rather than the technology or outcomes (six subclasses are suggested: food compound intake biomarkers (FCIBs), food or food component intake biomarkers (FIBs), dietary pattern biomarkers (DPBs), food compound status biomarkers (FCSBs), effect biomarkers, physiological or health state biomarkers). The application of this scheme is described in detail for the dietary and health area and is compared with previous biomarker classification for this field of research.

## Background

Biomarkers in general are objective measures used to characterise the current condition of a biological system. Many definitions exist, usually aimed at specific branch of science, e.g. medical therapeutics or nutrition. For instance, a working group under the US National Institutes of Health defined biomarkers as ‘a characteristic that is objectively measured and evaluated as an indicator of normal biological processes, pathogenic processes, or pharmacologic responses to a therapeutic intervention’. On the other hand, authors in the nutrition field have classified biomarkers as ‘a biochemical indicator of dietary intake/nutritional status (recent or long term), or an index of nutrient metabolism, or a marker of the biological consequences of dietary intake’ [1, 2]. A consensus statement from a Hohenheim conference on biomarker definitions in nutrition resulted in a definition of biomarkers as ‘test results related to exposure, susceptibility or biological effect’ [3]. An important characteristic of any biological system is that it is dynamic, involving life processes such as nutrient input, waste excretion, growth, movement, energy throughput, reproduction, annual cycles, aging, etc. The dynamic nature of biological systems is also the reason why objective characterization of the state of a biological system is needed. This is also a prerequisite for understanding how the system may respond, but also a useful tool to decide on the need for intervention if the system is moving into an undesired state such as disease.

Due to the large number of biomarker applications as well as the diversity of biomarker characteristics, there have been a number of published nutritionally relevant biomarker classification schemes (Table 1) [3–8], each using one specific characteristic of the biomarker as a criterion. However, none of them creates a truly universal classification without considerable ambiguity. This is because the same biomarker measurement, i.e. of a nutrient level, metabolite flux or other biological activity, may unintentionally end up in several classes, depending on their use in different studies.

**Table 1:** Published biomarker classification schemes

Criterion	Classification	Definition	Examples		References
			Sample type	Biomarker	
Temporal relationship with dietary intake	Short-term biomarkers	Biomarkers that respond to dietary intake within hours	Breath	Hydrogen (lactose intolerance)	[4]
			Plasma	^13^C–glucose (lactose intolerance)	
			Serum	Vitamin C (postprandial spikes)	
			Serum	Triglycerides (postprandial spikes)	
	Medium-term biomarkers	Biomarkers that respond to dietary intake over weeks or months	Red blood cell	Essential fatty acid (average of the previous 120 days of intake of essential fatty acids) Folate (average of the previous 120 days of intake of folate)	
	Long-term biomarkers	Biomarkers that respond to dietary intake over several months or years	Hair Toenail	Trace element (long-term intake of a trace element, e.g. Se)	
Relevant functional outcomes	Markers of exposure to a food compound	Markers that are related to the exposure to the food compound being studied, such as a serum, fecal, breath, urine or tissue marker	Red blood cell Blood	Folate (exposure to folate in food) Tryptophan (exposure to tryptophan in food)	[5]
	Markers of target function/biological response	Markers that are related to the target function or biological response such as changes in body fluids, levels of a metabolite, protein or enzyme or changes in a given function	Plasma Physical	Reduction of homocysteine (response to dietary folate) Blood pressure (response to dietary caffeine)	
	Markers of intermediate endpoint	Markers that are related to an appropriate intermediate endpoint of an improved state of health and well-being or reduction of risk of disease, or both, such as the measurement of biological processes that relate directly to the endpoint	Physical Bone	Extent of narrowing of the carotid artery (cardiovascular disease) Mineral density (risk of bone fracture)	
Association with intake	Recovery biomarkers	Biomarkers based on recovery of certain food compounds directly related to intake and not subject to substantial inter-individual differences	Urine Urine Urine	Doubly labeled water (metabolic rate and total energy expenditure) Nitrogen (protein intake) Potassium and sodium	[6, 8]
	Predictive biomarkers	Biomarkers that are sensitive, time dependent and show a dose-response relationship with intake levels but their overall recovery is lower than recovery biomarkers	Urine	24-h sucrose and fructose (sugar intake)	
	Concentration biomarkers	Biomarkers whose concentrations do correlate with intakes of corresponding foods or nutrients but the strength of the correlation is often lower (< 0.6) than that expected for recovery biomarkers (> 0.8)	Serum Blood	Vitamins (vitamin intake) Lipids (lipid intake)	
	Replacement biomarkers	Biomarkers that are closely related to concentration biomarkers and refer specifically to compounds for which information in food composition databases is unsatisfactory or unavailable	Urine Serum Urine	Aflatoxin Isoflavonoids and lignans (phytoestrogen intake) Phytoestrogens (phytoestrogen intake)	
Biological endpoint	Biomarker of exposure	Accurately reflecting intake/exposure	Any biological specimen	Plasma vitamin C	[3]
	Biomarker of susceptibility	Accurately reflecting (an aspect of) susceptibility	Any biological specimen	Low plasma vitamin C (risk of scurvy); high serum cholesterol or blood pressure (susceptibility to myocardial infarction); low bone mineral density (susceptibility to fractures)	
	Biomarkers of effect and efficacy	An established biomarker of efficacy is an indicator of an improvement of a physiologic function or a decrease in risk factors for a disease (it follows that effect biomarkers would also include the corresponding null or negative outcomes)	Any biological specimen	Changes in: serum cholesterol; blood pressure; bone formation, resorption or density; prostate specific antigen	
Purpose of the study	Biomarkers of dietary exposure	Biomarkers that are aimed at assessing dietary intake of different foods, nutrients, non-nutritive compounds or dietary patterns (recovery biomarkers, concentration biomarkers, recovery biomarkers and predictive biomarkers)	Urine	Nitrogen (protein intake)	[7]
	Biomarkers of nutritional status	Biomarkers that reflect not only intake but also metabolism of the nutrient(s) and possibly effects from disease processes	Plasma	Homocysteine (folate deficiency, one-carbon metabolic processes)	
	Biomarkers of health/disease	Biomarkers related to different intermediate phenotypes of a disease or even to the severity of the disease	Plasma Plasma	Total cholesterol (cardiovascular diseases) Triglycerides (cardiovascular diseases)	

**Fig. 1:**
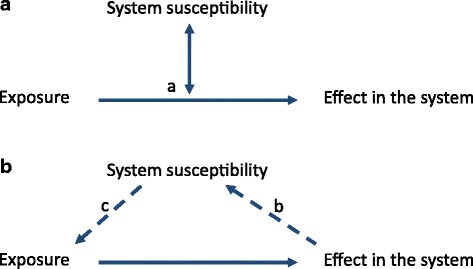
Interactions between the environment and a biological system. The system can be any organism or group of inter-dependent organisms and the environmental exposure can be any changes of the environment. The image in **a** applies to the static part of susceptibility and in **b** applies to the variable part of susceptibility. (*a*) Basic relationship between exposures, effects in a biological system and the susceptibility factors characteristic of the system. Susceptibility is basically an effect modifier for how the exposure(s) affect the biological response. (*b*) The effect imposed upon the biological system may eventually change the system characteristics thereby changing its susceptibility. (*c*) The exposure of the system may also be directly affected by the system susceptibility factors themselves, e.g. if exposure is avoided or exacerbated (e.g. if the sensation of hunger is increased so food intake increases beyond needs)

To reach a classification scheme for biomarkers with less ambiguity, it is necessary to understand the basic conditions affecting any potential biomarker in a biological system. Any organism can be recognised as a system that is constantly exposed to stimuli from both external and internal environments. These systems are therefore constantly influenced by a variety of factors and these exposures result in biological responses, depending on system susceptibility (Fig. 1).

Biological systems are dynamic and biomarker identification or biomarker measurements are therefore complicated by the intrinsic system’s response, which is aimed at reverting the system to the pre-challenge state (i.e. the host system response). If the system is unable to adjust, permanent changes may ensue, sometimes in an unpredictable way that may cause injury or disease [9]. The parameters that can be utilised to monitor and evaluate system states or changes are recognised as biomarkers.

**Fig. 2:**
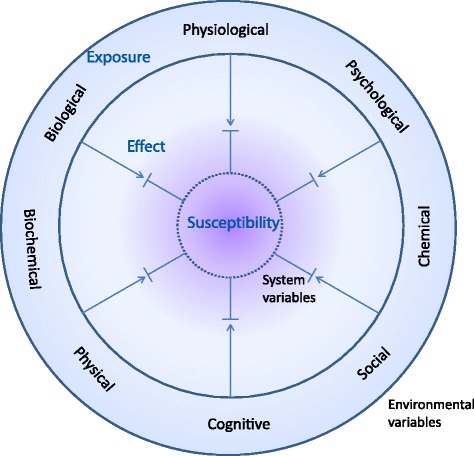
Diversity of interaction between the biological system with intrinsic system variables and the surrounding environmental variables. Both exposures (environmental variables) and their corresponding host susceptibility factors (intrinsic variables) are diverse in nature and the steady state level of effect biomarkers (measured as changes in system variables) in a balanced health situation reflects environmental stress that does not overtly challenge the system susceptibility

For human beings, as with most biological entities, there are three important interactions to consider between the individual and its environment: exposure, individual susceptibility and effect (Fig. 2). Exposures include physical, chemical, biochemical, biological, physiological, cognitive, psychological and social factors [10, 11]. They include external and visible factors such as foods, medicine and smoking, as well as less tangible factors like physical or psychological stress and societal inputs.

**Fig. 3:**
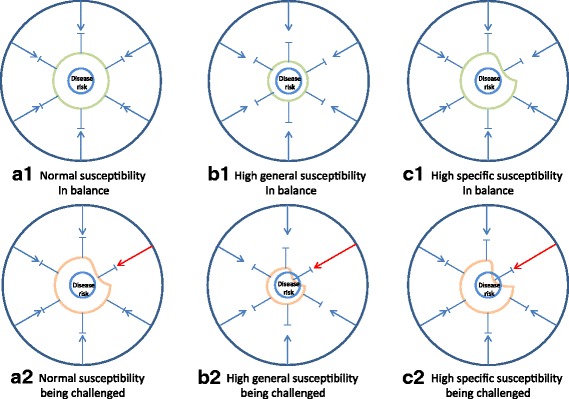
Balance and stress in a biological system. Any biological system including human individuals may experience periods of balance (**a1**, **b1** and **c1**) and periods of increased stress (**a2**, **b2** and **c2**). Systems with different susceptibility have different risk of developing disease when exposed to the same stress. For a system with normal (moderate or low) susceptibility (**a1**), an increased stress may be tolerated (**a2**) making the system come closer to disease risk but without causing disease. For a system with high general susceptibility (**b1**) or specific susceptibility (**c1**), an increased environmental challenge may overstep the system tolerance leading to imbalance and heightened risk of disease (**b2** and **c2**)

Susceptibility may be seen as an antonym to resilience affecting the ability to re-balance following a response to any of these exposures (Fig. 3). Individuals have static as well as variable elements as part of their intrinsic susceptibility or resilience. These are often termed as host factors.

The more static part of a biological system refers to the susceptibility that people are born with or grow up with, such as their genes, epigenetically encoded gestational factors, and often part of their cultural and social background. Other parts of the epigenome, culture, social factors and also the microbiota may be classified as semi-static since they may be changed to some extent [12, 13]. The variable part of a biological system refers to the consequence of exposures and cumulative effects, such as nutrition, fitness, immunity to infection, knowledge and mental balance. All of these are factors developed throughout life that can be changed gradually caused by impact or change of the environmental variables. Clearly, these static or variable factors also mutually interact to enhance or diminish their impact on the system. This fact underlines the point that susceptibility factors may not only act as modulators of exposures or effects but they also act mutually on each other. This further complicates the measurement of susceptibility.

Based on these interacting processes, any exposure may or may not lead to a series of changes in the system (e.g. in physical endurance, metabolism and intellectual capacity) which could lead to either a faster or slower aging processes or imbalances leading towards disease. All of these processes result in a complex, highly dynamic system, which is never in total balance. Biomarkers could, in theory, capture the state of all the on-going processes and changing balances, thereby giving a full characterization of the current state of the system, including health dynamics and disease risks. This should be seen in contrast to the current international consensus definition of health, which is more static. Under these consensus views, health is typically defined as a ‘state’ rather than a dynamic balance in all aspects of life [14]. Recent suggestions for a new ‘health’ definition support a more dynamic and operational assessment. In particular, health is now defined as an ability to cope with challenges in various dimensions of life [15]. Concepts relating to the measurement of health, i.e. biomarkers, should therefore also follow similar dynamics.

## Objectives of this review

There is widespread recognition that qualitative as well as quantitative changes in food intake can strongly affect the risk of diet-related disease. However, it is must also be acknowledged that (1) our current instruments for dietary assessment generate imprecise or even inaccurate estimates of intakes [16–18], (2) the short-term as well as the longer-term processes by which food components affect health are not fully understood [19, 20] and (3) individual static and dynamic susceptibility factors are not well described [21–23]. Therefore, it is necessary to develop robust and well-validated biomarkers to support the assessment of food intake and their effects at an individual level. The objective of this review is to provide an improved scheme for the classification and application of dietary and health biomarkers and to discuss the implications of this scheme’s use compared with previous schemes.

## Biomarkers for the dietary and health area

When it comes to dietary and health biomarkers, environmental exposure variables are often limited to the diet, i.e. the nutrients and all the non-nutritive components in foods. Non-nutritive components in this context may be largely inert or may, in analogy with established nutrients, affect health in a beneficial or adverse manner. Dietary and health biomarkers are focused on the measurement of these exposures and on quantifying the consequent biochemical, physiological, cognitive and biological changes affecting the exposed subjects. Many dietary intake biomarkers, at their current state of development, may be viewed mainly as validation tools for dietary assessment instruments (e.g. 24-h recalls, food diaries or food frequency questionnaires (FFQs) [24, 25]). In this sense, they may help to confirm the ‘nutritype’ of an individual, i.e. dividing a population into groups of common (typical) intake patterns. However, with the development of metabolomics, it is now possible to identify more food intake biomarkers and to provide a deeper understanding of metabolic dynamics. Dietary and health biomarkers may become central tools to get a better understanding of the association between diet, lifestyle or other environmental variables with individual disease risk [6]. In this case, dietary and health biomarkers may be defined as objective measures or indicators of food intake, the effects of dietary intake on the body and the consequent nutrition-related state of an individual or a group of individuals. Based on this definition, the dietary assessment instruments as such are not considered as biomarkers since they are not objective biological measures. However, these instruments are still the current best practice to assess dietary intake and are therefore used to support the discovery of potential dietary and health biomarkers. In analogy with biomarkers for clinical application, dietary and health biomarkers should be measured in suitable sample types which could capture the exposures or effects, and the kinetics of them should be well established for application and interpretation. Therefore, it is a prerequisite that the biomarkers meet a sufficient level of validation so that both analytical and biological aspects of the biomarker measurement methods are validated [26].

## Classification of dietary and health biomarkers

In general, dietary and health biomarkers can be subdivided into three major classes. These include exposure/intake, effect, or susceptibility/host factors (Figs. 1 and 2), as previously suggested by others [3].

- Exposure and intake biomarkers reflect the level of extrinsic variables that humans are exposed to, such as diets and food compounds, including nutrients and non-nutrients. They can usually be described in terms of rates of intake and concentrations in biofluids or tissues over a defined timeframe, e.g. for the single compounds, their kinetic parameters such as half-lives, total body burden or stores [4, 6].
- Effect biomarkers refer to the functional response of the human body to an exposure. They are defined in terms of the time course of response until they reach steady state or return to baseline levels. Effect biomarkers often integrate several challenges to reflect the effect of several extrinsic and intrinsic factors. Typical examples include changes in satiation, plasma glucose response and blood pressure [5].
- Susceptibility or host factor biomarkers represent the individual susceptibility or resilience to an exposure predicting the intensity of its effect on the individual. Susceptibility may be seen as the ‘background health status’, i.e. the sum of intrinsic or ‘host’ factors explaining current individual health-related risks. As already mentioned, they include static as well as variable susceptibility factors [3].

**Fig. 4:**
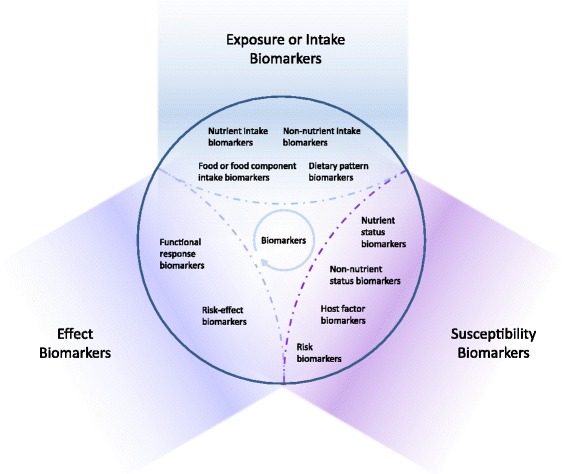
Classification of dietary and health biomarkers within the space shaped by the three hyper-categories of biomarkers, exposure, susceptibility and effect. See text for further discussion of the proposed subclasses of biomarkers. The interpretation of the measurement of a biomarker depends on its intended use

**Fig. 5:**
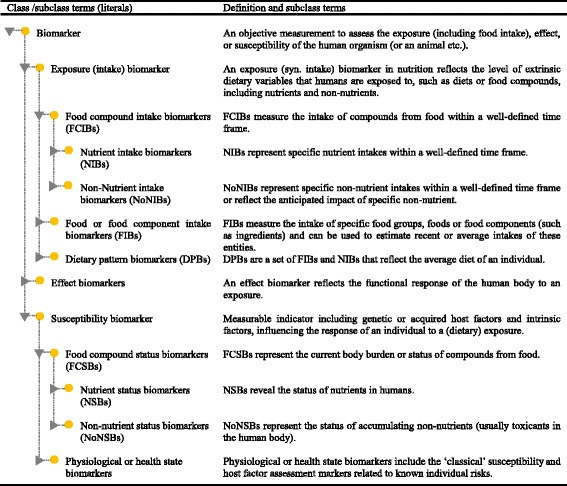
Proposed terms for initiating ontology for dietary and health biomarker

It is readily recognised that these classes overlap, just as with previous classifications. For instance, measurements of plasma glucose could be classified in any of the three classes, directly reflecting either a recent glucose intake (exposure), the glucose kinetics response (effect) or the ability to cope with a glucose challenge (susceptibility). Whether the measurement of a biomarker is classified into one class or another is, to a large extent, based on the intended application of the measurements. We would therefore like to define the three dietary and health biomarker classes as ‘hyper-categories of applications’ that may be divided into several subclasses as shown in Figs. 4 and 5 and in Table 2 below. These biomarker classes and subclasses share laboratory and clinical methodologies and incorporate most of the classes suggested in previously published classifications of dietary and health biomarkers.

**Table 2:** Prominent features and typical uses of the proposed subclasses of dietary and health biomarkers (none of these classes are exclusive in terms of the compounds measured)

Proposed classes	Proposed subclasses	Prominent features	Typical uses
Exposure (intake) biomarkers	1) Food compound intake biomarkers (FCIBs), divided into nutrient intake biomarkers (NIBs) and non-nutrient intake biomarkers (NoNIBs)^a^	- Specificity to chemically well-defined food compounds, e.g. nutrients or food-derived non-nutrients, such as bioactive compounds, including xenobiotics - Distinctive dose- and time-dependent responses after intake - May reflect acute or long-term intakes	Specific intake biomarkers for food compounds Intake biomarkers Exposure biomarkers
	2) Food or food component intake biomarkers (FIBs)^a^	- Specificity to particular foods, food components, or food groups - Distinct dose- and time-dependent responses after intake - A single metabolite or a combination of metabolites	Compliance biomarkers Markers of exposure to food components Biomarkers of dietary exposure Food intake biomarkers
	3) Dietary pattern biomarkers (DPBs)^a^	- A set of FCIBs and FIBs - Representation of ‘signal’ foods and nutrients in diets	Nutritype biomarkers Compliance biomarkers Markers of exposure to a dietary pattern
Effect biomarkers	4) Effect biomarkers, divided into functional response biomarkers and risk-effect biomarkers	- Indicators of response to a certain diet or dietary exposure - May include shorter-term or longer-term effects	Outcome biomarkers Efficacy biomarkers Impact biomarkers Functional response biomarkers Markers of target function/biological response
Susceptibility biomarkers	5) Food compound status biomarkers (FCSBs), divided into nutrient status biomarkers (NSBs) and non-nutrient status biomarkers (NoNSBs)	- Reflection of status for food compounds (nutrients and non-nutrients) - Nutrient status biomarkers are most often reflecting retained nutrients - Non-nutrient status biomarkers reflect cumulative intake of other food compounds, typically with long excretion half-lives - Accumulating xenobiotics may reflect potential toxicity - Indicators of susceptibility to nutrient or xenobiotic stress	Chronic exposure markers Biomarkers of nutritional status or susceptibility Biomarkers of non-nutrient status or susceptibility Biomarkers of body burden of toxicants
	6) Physiological or health state biomarkers, divided into host factor biomarkers and risk biomarkers	- Susceptibility markers - Assessment of host factors and disease risk - Host factors may be seen as individual variability - Health status biomarkers reflect current risk of disease or susceptibility to develop disease. Risks may be inborn or acquired - Potential or known indicators of susceptibility	Disease risk biomarkers Health state biomarkers Risk biomarkers Host factors Individual variability biomarkers Biomarkers of phenotypic trait Biomarkers of health/disease

^a^All three classes are intake biomarkers but differ with respect to the complexity of what the marker represents in the diet

## Discussion—dietary and health biomarker subclasses

The dietary and health biomarker classes proposed in Table 2 are meant to be a mutually exclusive list in the sense that any application of a dietary and health biomarker should be covered by only one of the classes. The basic concept is that the biomarker classification is determined solely by the purpose of using the biomarker and not by the assay as such. This is detailed below with a range of examples. Although the classification scheme builds upon an existing scheme, the division by study purpose rather than by assay methodology is conceptually novel for the diet-and-health field while it has been used more often in the clinical area; this classification provides a more strict language for the dietary and health biomarker area. Each biomarker class may be further subdivided as explained below and shown in Fig. 4.

**Fig. 6:**
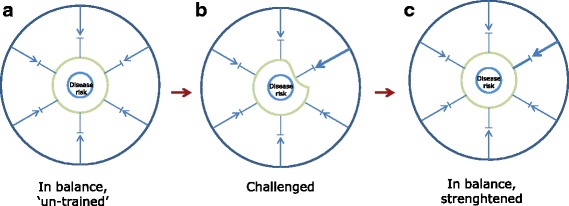
System training by challenging. **a** In the naive, untrained but balanced state, the capacity to withstand a challenge is limited. **b** An increased challenge intensity will offset the system causing a temporary, weaker state. **c** Following a biological response such as enzyme induction, formation of antibodies or muscle re-building, the system becomes more resilient to challenge or stress

1. Food compound intake biomarkers (FCIBs): The compounds in food may be nutrients or other chemical entities, i.e. non-nutrients. Nutrient intake biomarkers (NIBs) represent specific nutrient intakes within a well-defined timeframe as previously detailed by Potischman [4]. For instance, urinary potassium could be used to assess the dietary potassium intake over a collection period of around 24 h [25]. Biomarkers of long-term exposures may also be NIBs, for instance toenail selenium serves the purpose of representing the long-term (0.5–1 year) intake of selenium [27–29]. NIBs for long-term intakes should not be confused with the use of the same measurements to provide a status of current nutrient adequacy (i.e. as NSBs, see subclass 4 below). The NIBs typically fluctuate around an average reflecting the median intake over a period defined by their kinetics.
  Another FCIB subcategory would be the non-nutrient intake biomarkers, NoNIBs. NoNIBs may be further subdivided by exposure timeframe in analogy with the NIBs or according to their anticipated impact. For instance, biomarkers of zeaxanthin or resveratrol intake can be used to represent putatively beneficial non-nutrients, and biomarkers of lead, solanine or organophosphorous pesticide intake can serve as indicators of specific toxicants present in the diet. An ideal NoNIB has a zero value when the compound has not been present in the food or diet and increases significantly after intake with well-characterised kinetics. Many polar plant ‘secondary’ metabolites such as phenolics, simple terpenes and others follow this pattern [30]. Again, the NoNIB class should not be confused with NoNSB (subclass 4 below) where measurements of the same compounds are used to evaluate whether a safety limit is reached.
2. Food or food component intake biomarkers (FIBs): FIBs measure the intake of specific food groups, foods or food components (such as ingredients) and can be used to estimate recent or average intakes of these entities. They could provide objective assessment of dietary intake in nutrition research; therefore, they might be a promising tool to qualify or even substitute dietary assessment instruments. The timeframe represented by a FIB depends on the kinetics of the metabolite measured.
  The FIB could be a single compound biomarker (typically a NoNIB) or a combined biomarker. For instance, proline betaine excretion could be used as a biomarker of recent orange juice intake [31] or citrus fruit consumption [32, 33] and ethyl glucuronide in blood or urine could serve as a biomarker of alcoholic beverage consumption within the last 48 h [34–36]. More specific combined biomarkers have also been proposed. For instance, tartrate together with ethyl glucuronide could serve as a biomarker of red wine consumption [37]; four different beer constituents have been proposed as a multi-component biomarker of beer intake [38]; and ratios of specific alkylresorcinols have been suggested as biomarkers of wheat or rye fibers [39–41]. As seen from these examples, the addition of two or more FCIBs to form a combined FIB may be done in several ways (Table 3). Specifically, this may be done by including one of two or more FCIBs, by summing up signals from one or more similar metabolites, by calculating ratios of two FCIBs or by presenting a pattern of several FCIBs along with a rule for how much of this pattern should be covered. Whole food groups may be represented by several FIBs such as a combination of several flavonoids for fruit and vegetables [42] or by a single common characteristic compound as exemplified by proline betaine (citrus fruit) or ethyl glucuronide (alcoholic beverages).
  FIBs will be the subject of a series of reviews to be published in this current journal issue. Again, the ideal food intake biomarker is zero when the food is not ingested throughout a ‘wash-out’ period but is measurable showing distinct dose- and time-dependent responses after intake [30]. Those responses should, as far as possible, be independent of individual host factors, e.g. differences in metabolic or transport rates or in gut microbial functionality; therefore, food compounds that are not metabolised may be the most promising FIBs. Most of the FIBs discussed here except ethyl glucuronide have not been formally validated, and extra studies are needed to improve the validity of them [43].
3. Dietary pattern biomarkers (DPBs): DPBs are a set of FIBs and FCIBs that reflect the average diet of an individual. They can be used to distinguish between different dietary habits or to highlight the relative adherence to a pre-defined diet such as Mediterranean [44] or Nordic diets [45, 46]. Typically, these biomarkers represent a number of ‘signal’ foods and nutrients that are more abundant in a certain diet, e.g. biomarkers of olive oil, citrus fruits, greens, nuts and fish along with alpha-linoleic acid and specific polyphenol signalling Mediterranean-type diets. The biomarkers of such ‘signal foods’ may also be used in combinations to produce a score-like evaluation of the consumption of the pattern. They usually overlap to some extent with the foods and nutrients used to define the diet indexes from questionnaire data, such as the Healthy Eating Index HEI [47], or the Mediterranean diet score MDS [48]. Since most people eat a variety of foods in each meal but never eat all signal foods at the same time, it is the pattern rather than the single biomarkers that reflect the dietary signature. Intake biomarkers are often used to assess the relationship between diet and health effects. It is often difficult to evaluate an isolated effect of a single nutrient in a complex diet since there are interactions between nutrients and other dietary factors. The DPBs may be useful to assess the overall adherence to diets in longer-term studies of their health effects.
  To make good use of DPBs, 24-h urine samples and repeated sampling are preferred to eliminate the extraneous variability caused by variables such as the timing of sample collection and within-person variation [49, 50]. Three samples taken with several months interval might be sufficient to reflect long-term status [51], but the sample collection interval might depend on various factors such as season and the length of the study period [49]. For example, the consumption of fruit and vegetables varies according to seasons, which could lead to biased estimate of the habitual intake of some nutrients such as vitamin C [52]. In this case, sampling in every season might be necessary to obtain a more precise estimate of average diet.
4. Effect biomarkers are used to monitor changes in biochemical, physiological or psychological state as a response to nutritional exposures. These biomarkers may be divided into (a) those only indirectly associated with risk, i.e. most biomarkers related to a functional physiological or metabolic response (functional response biomarkers), and (b) those directly related to risk, i.e. risk-effect biomarkers describing an effect on an established risk biomarker (for risk biomarkers, see section 6b below). A functional response is often taken to indicate a certain mechanism of action while risk-related biomarkers additionally have a recognised cause-and-effect relationship to disease.

- Functional response biomarkers could be biomarkers of enzyme induction, satiety, endurance, gene expression, or an acute-phase inflammatory marker. Some functional change biomarkers cannot readily be interpreted. Great caution should be exercised when biomarkers are used that have not been fully validated for use as risk-related biomarkers. Sometimes, indicators of ‘effects’ with unknown biological consequence have gained widespread use although the measurements may actually be irrelevant with respect to disease risk. An example may be the antioxidant capacity of plasma, which is easy to measure and even reproducible but antioxidant capacity has so far never been shown to be associated with disease risk or to have any other biological consequence to humans [53–55]. In other cases, these biomarkers are related to both exposure and effect. This is the case for repair products resulting from adducts of reactive compounds with macromolecules, e.g. aflatoxin B1-deoxyguanosine adducts, sometimes termed ‘markers of target dose’ to indicate an acute response phase for these investigative biomarkers. Some effect biomarkers have well-established mechanisms and risk correlates but are not causally related to risk. Plasma C-reactive protein (CRP), a well-known acute-phase biomarker in inflammation, is a good example of such a functional response biomarker. CRP has also been clearly associated with stroke [56]. However, a dietary factor affecting CRP may not be relevant for modulating the risk of stroke since CRP is not on the causal pathway to ischemic stroke [57].
- Another group of effect biomarkers are those that are also used as classical biomarkers of risk (see section 6b), here termed risk-effect biomarkers. Used as effect biomarkers, they include changes in, e.g. lipoprotein ratios [58], blood pressure [59] or fasting plasma glucose levels [60]. Only a dynamic change, in these biomarkers, represents an effect, i.e. as a response to a challenge, a dietary change, a medical treatment, etc. This application of the measurements are most often seen in intervention trials where the focus is whether a certain treatment could potentially affect a disease risk; in this capacity, the risk-effect biomarkers are usually applied as surrogate markers of the potential to alter a certain disease risk. For example, the changes in total, LDL and HDL cholesterol could be used to evaluate the hypocholesterolemic effects of some bioactives, as in the case of β-glucans [61] or even by whole food groups as in the case of fruit and vegetables [62]. The changes in the risk-effect biomarkers could also be used to evaluate the potential effect on disease risk reduction by following certain dietary patterns. For instance, change in blood pressure after intake of Mediterranean diets during a 4-year period has been used to investigate the potential mechanism for change in risk of cardiovascular disease [63]; also in a 6-month intervention trial, the changes in fasting plasma glucose, fasting serum insulin and HOMA-IR were measured to estimate the effect of a defined Nordic diet on the risk of diabetes [64]. These same measurements may also be used to characterise a health status or risk in a more static sense, e.g. as a baseline characteristic in a nutrition trial and in this case belongs to biomarkers of susceptibility (see 6b below).

5) Food compound status biomarkers (FCSBs) represent the current body burden or status of compounds from food. These compounds may be nutrients that are actively absorbed and retained or they may be non-nutrient compounds, including toxicants, which are able to build up higher concentrations in the body because they are lipophilic or otherwise difficult to clear from the body.
  In the case of nutrient status biomarkers (NSBs), they reveal the status of nutrients in humans, such as a micronutrient sufficiency or deficiency. For instance, ferritin could be used as a sensitive indicator of iron stores [65, 66]; serum cobalamin levels can be used to detect vitamin B12 deficiency [67] and red blood cell glutathione peroxidase can be used to assess current selenium status [29, 68, 69]. The major difference between NIBs and NSBs is their use; when a biomarker is applied for assessing nutrient intakes, it is a NIB. When the same biomarker measurement is used to assess current nutritional status, it is a NSB. NSBs assess potential vulnerability or healthiness, which is part of the variable susceptibility that indicates closeness to adequacy, deficiency or overload.
  Likewise, in the case of non-nutrients, the analogous NoNSBs represent the status of accumulating non-nutrients (usually toxicants in the human body) and therefore a cumulative exposure or risk. The measurement of halogenated organics and heavy metals themselves could serve as an indicator of their accumulation in the body over time. For example, cadmium can be accumulated in the kidneys, reflecting not only long-term exposures but also cadmium-related disease risk. Urinary cadmium therefore could be used to assess the cumulative cadmium intake and current body burden [70]. Since biomarkers of toxicant body burden may be seen as analogous to NSBs, they are susceptibility-related phenotypic biomarkers as long as they are used to measure status in comparison with an intake limit or to evaluate ensuing health risks. If the same compounds are measured to estimate average long-term exposure, they would be classified as NoNIBs. Some hydrophobic non-nutrients such as lutein and lycopene may also accumulate in the body; lutein is not considered a nutrient and has not so far been approved for health claims by EFSA [71]. However, lutein in combination with certain nutrients has documented effects on age-related macular degeneration following medical use [72] indicating that lutein levels, particularly in the eye, may serve as a NoNSB. Plasma lycopene has not yet been shown unequivocally to have an effect on health [73]; however, lycopene has been considered as a biomarker for cumulative intake of tomato products, since tomatoes are one of its main sources in the diet [74, 75], thereby showing potential as a NoNIB.
6) Physiological or health state biomarkers include the ‘classical’ susceptibility and host factor assessment biomarkers related to known individual health risks. A host factor may be considered a personal intrinsic quality or trait influencing susceptibility to disease—or resilience. As described above, assessment of host factors or risk sometimes use exactly the same procedures or assays as those used for determining an effect of an intervention. However, here they are used as biomarkers to characterise the individual or a group with respect to a functional characteristic or to the susceptibility to develop disease. These biomarkers represent therefore the variability between individuals with respect to health as well as with respect to disease risk. The classification of these biomarkers is complicated by the fact that some physiological or health state biomarkers are measured as a response to a standard (dietary) challenge, e.g. the oral glucose tolerance test (OGTT). The OGTT is therefore a response measurement used to characterise an individual’s current health state or disease risk. In analogy to the division of effect biomarkers into biomarkers for a change in functional response and biomarkers affecting risk, physiological or health state biomarkers may also be subdivided into (a) host factor biomarkers and (b) risk biomarkers.

- Host factor biomarkers encompass a large number of status biomarkers and cannot (yet) be used to predict risk. Some are static, for instance, genotypes represent one of the most static host factors of living organisms. Obviously, it is only the known functional gene variants (or haplotype markers) that may be used as host factor biomarkers. Mutations do occur but they are random while other host factors may change over time in a predictable manner as a functional response to exposures, challenges or treatments as described for the effect biomarkers above (section 4). This functional response may actually change the susceptibility so that a more adequate response to the challenge is ‘learned’, see Fig. 6. Good examples of altered susceptibility ‘learned’ by functional responses are acquired immunity, exercise-improved fitness, and acquired tolerance to poison by enzyme induction. Host factors with an ability to be altered by challenges include nutritional, metabolic, epigenomic, microbial, immunological and physiologic phenotypes. These may form complex relationships with risks. For example, in metabolomics, we can observe hundreds of metabolites indicating nutritional status, mostly within normal levels. Collectively, they seem to be a characteristic of each individual, i.e. a metabolic phenotype reflecting an individual’s current metabolic abilities and resulting in clustering of repeated metabolic profiles from each individual studied [76, 77]. An example from genetics is that the ability of fast or slow metabolism of caffeine is not directly predicting effects on sleep. Homozygotic carriers of either allele may be equally affected in their ability to sleep after a cup of coffee since this is more strongly determined by a polymorphism in the genes encoding the caffeine sensitivity of the adenosine A2A receptor in the brain [78]. So the latter polymorphism is the most important host factor determining sleeping ability. The former may be more important to predict the blood pressure response to caffeine intake [79]. Both polymorphisms represent (static) host factors but the latter may also be a risk modulator related to myocardial infarction [80]. The impact of these two host factors on risk is still unclear so they are not established risk biomarkers.
- In contrast, risk biomarkers are normally used to predict specific aspects of an individual’s disease risk or development. They can be graded on a scale from altered susceptibility to disease diagnostics or prognostics. These susceptibility biomarkers are most often measured in a cross-sectional or individual setting. Specifically, they are used to characterise risk at baseline in a population or they may be used individually using a sample collected at a medical practitioner’s office to determine whether a treatment should be instituted or altered. Biomarkers like systolic blood pressure, OGTT or fasting glucose levels in serum or plasma are good examples of disease risk/diagnostic biomarkers with clear international guidelines for how to interpret readings from an individual and on how and when to start treatment [81, 82]. Other risk biomarkers, such as low insulin sensitivity or age, also have well-described relationships with risk of disease, and combined risk scores including several susceptibility biomarkers are issued by organizations such as the American Heart Association [83]. Another more complex and explorative example is a biomarker predicting increased breast cancer risk composed of a combination of questionnaires, metabolomics and physiological measurements [84]. Such disease risk patterns from combined data are putative risk biomarkers but need rigorous validation.

**Table 3:** Examples of complex FIBs using combination of FCIBs

Food or food component	Combined FIB	Modes for combining FCIBs				Source of information
		Either-or	Sum of 2 or more	Pattern^a^	Ratios of biomarkers	
Beer	Isoxanthohumol/8-prenylnaringenin	X	X			[85]
Soy	Equol/O-desmethylangolensin	X	X			[86]
Beer	N-methyl tyramine sulfate, the sum of iso-α-acids and tricyclohumols, pyro-glutamyl proline, 2-ethyl malate			X (3/4)		[38]
Red wine	Tartrate, ethyl glucuronide			X (2/2)		[37]
Sugar-sweetened beverages	Formate, citrulline, taurine, isocitrate			X (4/4)		[87]
Wheat or rye fibers	Ratios of specific alkylresorcinols (C17:0/C21:0)				X	[39–41]
Fruit and vegetables	Ten flavonoids		X			[42]
Walnut	Metabolites of fatty acid metabolism (10-hydroxy-decene-4,6-diynoic acid sulfate; tridecadienoic/tridecynoic acid glucuronide), ellagitannin-derived microbial compounds (urolithin A glucuronide; urolithin A sulfate), and intermediate metabolites of the tryptophan/serotonin pathway (3-indolecarboxylic acid glucuronide)			X (5/5)		[88]
Coffee	Atractyligenin glucuronide, cyclo(isoleucylprolyl), 1-methylxanthine and trigonelline		X			[89]

^a^Supplemented with the rule for how many FIBs in the pattern should be covered

## Conclusion

The current pace of dietary and health biomarker discovery and application is higher than ever before due to the rapid development of ‘omics’ technologies and ‘big data’ techniques. As a result, this area can be defined as frontier research shaping the development of many important tools for future research in nutrition and health. Several frameworks for naming and classifying biomarkers exist. One of them is the common overall division into exposure, effect and susceptibility biomarkers. However, this division has been previously described as very static, resulting in a need for continuous updates due to the rapid developments in technologies and applications. There is now an urgent need for the classification of biomarkers to be far more flexible. This is because the actual laboratory or clinical measurement provided for any biomarker is not directly linked with its use to measure exposure, effects or susceptibility. The same assay procedure may be used for all of these purposes, so we believe it is the intended use that best determines the classification of a given biomarker. This concept provides a much improved and far more flexible classification scheme for dietary and health biomarkers that nicely fits into the current complex scenario of research in the nutrition and health area.
